# Comparing quantitative image parameters between animal and clinical CT-scanners: a translational phantom study analysis

**DOI:** 10.3389/fmed.2024.1407235

**Published:** 2024-06-05

**Authors:** Abhinay Vellala, Carolin Mogler, Florian Haag, Fabian Tollens, Henning Rudolf, Friedrich Pietsch, Carmen Wängler, Björn Wängler, Stefan O. Schoenberg, Matthias F. Froelich, Alexander Hertel

**Affiliations:** ^1^Department of Radiology and Nuclear medicine, University Medical Centre Mannheim, University of Heidelberg, Mannheim, Germany; ^2^Department of Pathology, Technical University of Munich, Munich, Germany

**Keywords:** radiomics, photon counting CT, animal models, translation, diagnostic integration

## Abstract

**Purpose:**

This study compares phantom-based variability of extracted radiomics features from scans on a photon counting CT (PCCT) and an experimental animal PET/CT-scanner (Albira II) to investigate the potential of radiomics for translation from animal models to human scans. While oncological basic research in animal PET/CT has allowed an intrinsic comparison between PET and CT, but no 1:1 translation to a human CT scanner due to resolution and noise limitations, Radiomics as a statistical and thus scale-independent method can potentially close the critical gap.

**Methods:**

Two phantoms were scanned on a PCCT and animal PET/CT-scanner with different scan parameters and then the radiomics parameters were extracted. A Principal Component Analysis (PCA) was conducted. To overcome the limitation of a small dataset, a data augmentation technique was applied. A Ridge Classifier was trained and a Feature Importance- and Cluster analysis was performed.

**Results:**

PCA and Cluster Analysis shows a clear differentiation between phantom types while emphasizing the comparability of both scanners. The Ridge Classifier exhibited a strong training performance with 93% accuracy, but faced challenges in generalization with a test accuracy of 62%.

**Conclusion:**

These results show that radiomics has great potential as a translational tool between animal models and human routine diagnostics, especially using the novel photon counting technique. This is another crucial step towards integration of radiomics analysis into clinical practice.

## Highlights

- Radiomics bridges animal and human scans.
- Photon counting CT enhances translational potential.
- Radiomics aids clinical diagnostic integration.

## Introduction

A significant portion of CT imaging is conducted on patients with oncological conditions. These patients undergo regular follow-up examinations to monitor the status of their cancer and make any necessary adjustments to their oncological treatment regimen (1, 2). However, CT imaging has limitations in differentiating between tissue types, particularly in oncological imaging.

Positron Emission Tomography combined with Computed Tomography (PET/CT) has emerged as a powerful and integral tool in oncological imaging. This hybrid imaging modality seamlessly integrates functional and anatomical information, offering clinicians unparalleled insights into the metabolic activity, receptor status, tissue function and precise localization of tumors within the body (3, 4). Despite its numerous advantages, PET/CT imaging is not without limitations. One notable constraint lies in its relatively high cost, limiting widespread accessibility. Additionally, the spatial resolution of PET/CT may be suboptimal for detecting small lesions, necessitating complementary imaging techniques for a more comprehensive evaluation in certain cases (5, 6).

Due to these limitations, regular CT imaging continues to be the imaging modality of choice when it comes to monitoring the progress of oncological diseases, even if the functional information of nuclear medicine imaging, which can provide further information on tumors and their activity, is missing. At least about the assessment of the textural properties of various tumors, there are approaches in CT imaging to generate more information, for example by extracting radiomics parameters.

Radiomics has emerged as a promising approach to improve lesion classification in oncological imaging (7–9). It involves the high-throughput extraction of quantitative features from medical images that can provide valuable information about the textural properties of a region of interest and possible tumoral heterogeneity, which potentially has a big impact on personalized oncological treatment (10–12). However, concerns about comparability and radiomics feature stability hamper the translation of results from preclinical scanners to clinical scanners (13–15).

The emergence of photon counting CT (PCCT), a new imaging technique that directly measures photons without a traditional energy-integrating detector, has created new opportunities for radiomics research (16, 17). This technology enables the evaluation of radiomics features’ stability in CT imaging, which is crucial for developing robust radiomics models for clinical use (18, 19).

While in basic oncological research investigations are carried out on animal models in special animal PET-CT scanners and an intrinsic comparison between PET and CT is possible, a 1:1 translation to a human CT scanner is not possible due to noise and resolution limitations. Radiomics leverages statistical methods to extract and analyze quantitative features from images, making it a robust tool for medical image analysis. The use of normalization and standardisation processes ensures that the extracted features are independent of scale and other imaging parameters, enabling consistent and comparable results in different environments and therefore has, in the context of experimental animal CT scanners and novel human CT scanners, great potential to enable better translation.

The purpose of this study was to assess the variability of radiomics features of phantom scans on a research-oriented animal CT scanner and a clinical photon counting CT. This study aimed to investigate the consistency of radiomics features across different CT scanners and the potential of radiomics as a tool for a scale-independent translation between experimental and clinical scanners such as the PCCT.

## Materials and methods

### Data acquisition

Two fruits, a mandarin and a plum, were selected and scanned using the Albira II PET/SPECT/CT Imaging System (Bruker) and Photon Counting Computer Tomograph Naeotom Alpha (Siemens). These specific fruits were chosen based on two criteria: (1) their ability to fit within the compact gantry of the Albira II scanner and (2) their distinct biological structures, which would allow for the generalization of findings to more heterogeneous samples. Prior to scanning, both fruits were meticulously inspected for defects to ensure integrity. Two fruits of nearly identical size were chosen for the study. They were placed adjacent to each other on the examination couch and secured with adhesive tape to maintain consistent positioning. Both fruits were scanned with each scanner/protocol simultaneously to ensure maximum comparability between phantoms and modalities. The fruit phantoms were first scanned on the Photon Counting CT and then immediately afterwards on the Albira II without any time delay in order to avoid a possible change in the fruit over time.

The Albrira Imaging System, developed by Bruker, was employed for the data acquisition process. The system offers a maximum resolution of 2400×2400 pixels and a field of view (FOV) measuring 70×70 mm (transaxial × axial). Two different dose and voltage settings were used during the measurements. The high dose/voltage configuration involved a current of 400 μA and a voltage of 45 kV, while the low dose/voltage configuration utilized a current of 200 μA and a voltage of 35 kV. These settings were selected to capture the internal structures of the fruits at different levels of detail. Table 1 shows a comparison of the technical data of the two scanner types used. Several scans were carried out with different protocols: “Good” and “Best” mode with 35 kV and 0.2 mA and 45 kV and 0.4 mA respectively, as well as a “High Resolution” mode with 35 kV and 45 kV, respectively, (the protocols are predefined as such by the manufacturer Bruker; the exact scan parameters can be found in Table 2).

**Table 1:** Scanner hardware applied for the study.

	Albira II PET/CT	Siemens NAEOTOM Alpha
Detector	Energy integrating detector	Photon counting detector
In-plane resolution	50 μm	100 μm
Scanning time	20 min	<1 s
Dose	Up to 1,000 mSv	<1 mSv
Tube voltage modes	35/45 kV	70/ … / 140 kV
Scanning modes	Standard, High-Res	Standard, High-Res

**Table 2:** Scan parameter of the Albira II scan protocols “Good,” “Best,” and “HR.”

Settings	Projections	Total exposure time	Voxel size
Good	400	5	250 μm
Best	600	8	125 μm
High resolution	1,000	10	35 μm

The acquired imaging data were converted into the Digital Imaging and Communications in Medicine-format (DICOM) using the PMOD software. PMOD [Version 4.4, PMOD Technologies LLC, Zurich, Switzerland (20)] is a tool from Bruker with a variety of functions for image post processing, image analysis and quantification. The functions include multimodal image analysis, quantitative assessment, region-of-interest (ROI) analysis, dynamic image evaluation, image processing and enhancement, data management and export, as well as a user-friendly interface. DICOM conversion facilitated standardized storage, transmission, and analysis of the imaging data. The reconstructed images were generated using the filtered backprojection (FBP) algorithm. FBP is a widely used technique for reconstructing images from X-ray projection data. It calculates the attenuation of X-rays passing through the object and generates a two-dimensional representation of the internal structures. The FBP algorithm was applied to the acquired data to reconstruct the images of the Plum and Mandarin samples.

On the PCCT, a standard Abdomen protocol was used. The in-plane resolution of the scanner is 1 mm with a tube voltage of 120 kV with an effective tube current of 7mAs. The acquired images were exported as DICOM-Files.

### Radiomics feature extraction

The following workflow was used for the extraction of radiomics features from the CT images. Semi-automatic segmentation of the phantoms was performed using MITK Workbench (v2022.10, German Cancer Research Centre, Heidelberg, Germany). The segmentation process was performed by a radiological resident with more than 4 years of experience. The segmented images were exported in a compressed Neuroimaging Informatics Technology Initiative format (nifti). The images were then loaded into a Docker container that was created based on PyRadiomics (Version 3.0.1), an open-source software library for radiomics feature extraction (21).

A total of 1,022 radiomics features were extracted. The feature extraction process was performed using default settings for PyRadiomics and complies with the Image biomarker standardisation initiative (IBSI) (22). The feature extraction included gray-level discretization with a fixed bin width with 32 bins, image resampling to a voxel size of 1 mm^3^, and a mask dilation radius of 1 voxel. The extracted radiomic features include those from the following feature families: first-order statistics (FO), shape-based (SH), gray level co-occurrence matrix (GLCM), gray level run length matrix (GLRLM), gray level size zone matrix (GLSZM), neighboring gray tone difference matrix (NGTDM), and gray level dependence matrix (GLDM). Re-segmentation was not applied in this study. The segmentation masks used for feature extraction were directly applied without any further modification or re-segmentation to ensure that the features were extracted from the original segmented regions of interest.

### Principal component analysis

**Figure 1:**
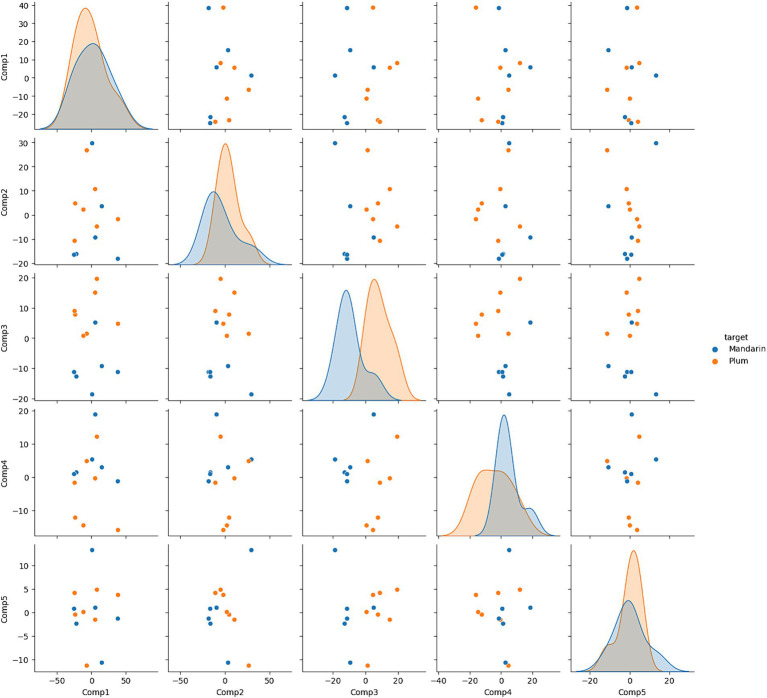
Pairplot between principal components. The plot shows the difference in densities of the phantoms.

Principal Component Analysis (PCA) is used to reduce the dimensions of extracted radiomics features. The 1,022 features are reduced to 2 components and gradually increased to 5 components until 100% variance is explained to understand the data. Sklearn package in python is used to apply the PCA method on the data. PCA analysis proved to show the difference between the mean of two phantoms. Figure 1 shows the pair plot of each phantom for all components. The plot clearly shows the difference of mean between the density of each phantom.

### Data synthesis

To address the constraint of a limited dataset, a data augmentation technique was implemented. The process involved synthesizing additional data points by using statistical properties, specifically mean and standard deviation, from two distinct classes (plums and mandarins). This resulted in the expansion of the dataset to 500 rows per class. The aim of this data synthesis was to provide a more extensive training set, enabling the model to capture a broader spectrum of patterns and variations present in the data.

### Ridge classifier

For the binary classification task, a Ridge Classifier was chosen for its suitability in handling such tasks (23). The model underwent training using the augmented dataset. This involved optimizing parameters and coefficients to establish a decision boundary between the two classes. Notably, the model demonstrated adaptability to the augmented dataset during the training process.

### Clustering analysis

Simultaneously, agglomerative clustering was applied to the original dataset. This unsupervised learning technique grouped data points based on their intrinsic similarities, forming hierarchical clusters. Notably, the clustering analysis aimed to reveal underlying patterns within the data. The results were particularly insightful in identifying distinct clusters associated with the specific CT scanner used during data collection, indicating the impact of scanner variability on the dataset.

## Results

### Phantom scans

**Figure 2:**
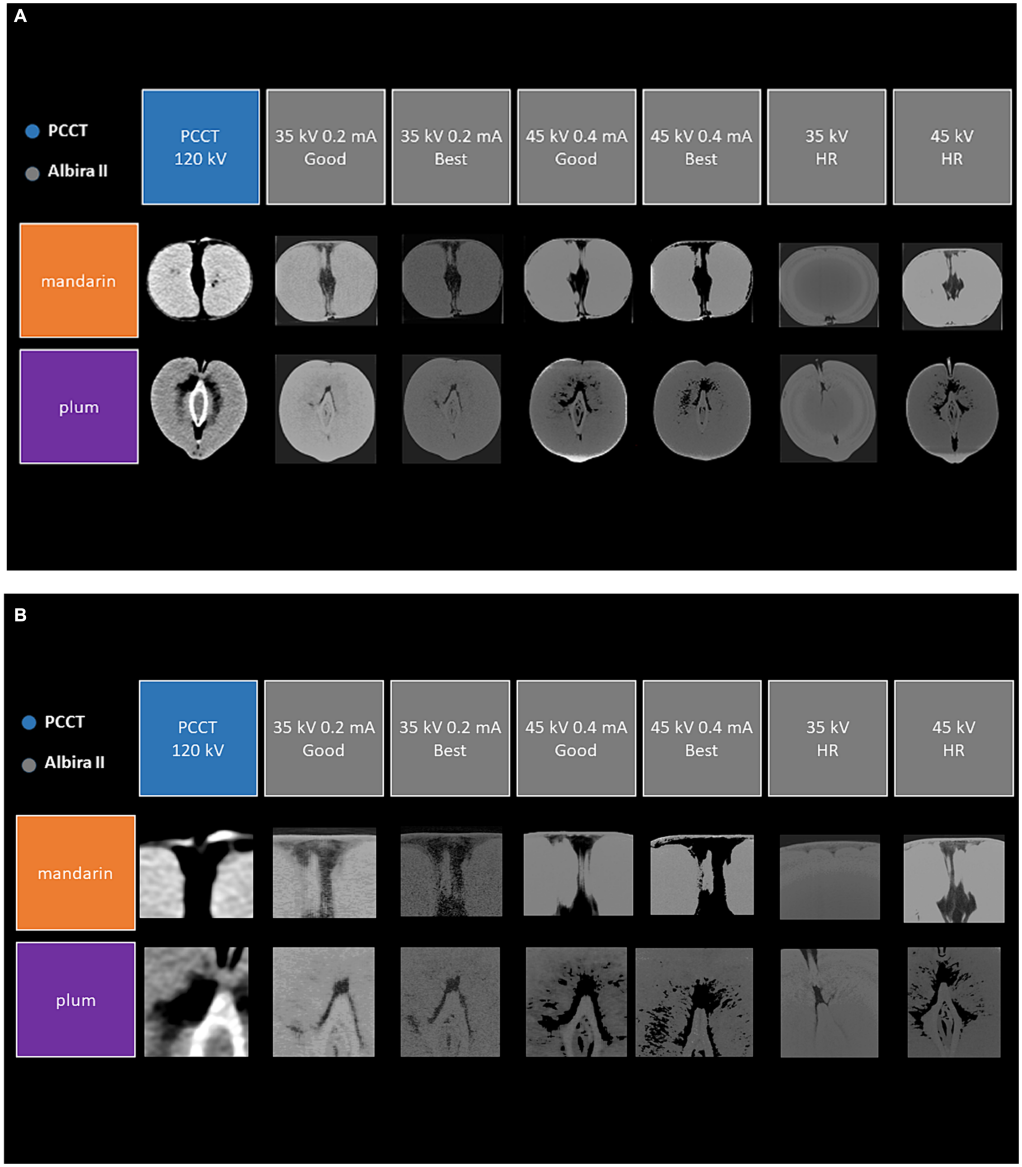
Coronal view of the scanned phantoms (mandarin and plum) with different CT scanners and scan protocols **(A)** and zoomed in images **(B)**.

The plums and mandarins were scanned several times in the animal PET/CT scanner with different scan settings as described above. These different scan protocols were chosen to obtain images with varying levels of clarity and detail. PCCT Scans were performed with the scan parameters listed above. Scan duration was approximately 10 s. Dose used for the phantom scan was 17,3mGycm (DLP). Figure 2A shows a comparison of the scanned phantoms in the coronal slice plane depending on the different scan parameters. Figure 2B shows them zoomed in for a better comparison of the textural resolution. In particular, the images of the small animal PET/CT scanner with the settings 45 kV 0.4 mA in “Best” mode and 45 kV in “HR” mode show an extremely high resolution with regard to the fine textural properties of the phantoms in the area of the central core. The scans of the PCCT show a comparable resolution to the 35 kV scans of the animal scanner and better resolution than the 35 kV “HR” mode, but with a significantly shorter scan time and considerably lower radiation dose.

### Phantom detection

In-depth comprehension of the dataset is attained through Principal Component Analysis (PCA). Five components capture 99% of the explainable variance. The density plot of the two classes in Figure 1 distinctly illustrates a disparity in means across all components. To support this observation, a *T*-test yields a test statistic of 0.027, providing statistical evidence of the significant mean difference between the two classes.

The Ridge Classifier, after hyperparameter tuning with the solver set to “lsqr” and a tolerance (tol) of 0.3, exhibited strong performance during training, achieving an accuracy of 93%. However, the model’s test accuracy was lower at 62%, indicating some challenges in generalizing to unseen data. Notably, the model demonstrated the ability to differentiate between the two classes with an F1 score of 0.6 on the test data, highlighting its effectiveness in capturing both precision and recall.

In addition to the classification results, a plot illustrating the feature importance of the Ridge Classifier provides valuable insights into the variables contributing significantly to the model’s decision-making process. This feature importance plot aids in the interpretation of the model’s decision boundaries and identifies key factors influencing its predictions.

**Figure 3:**
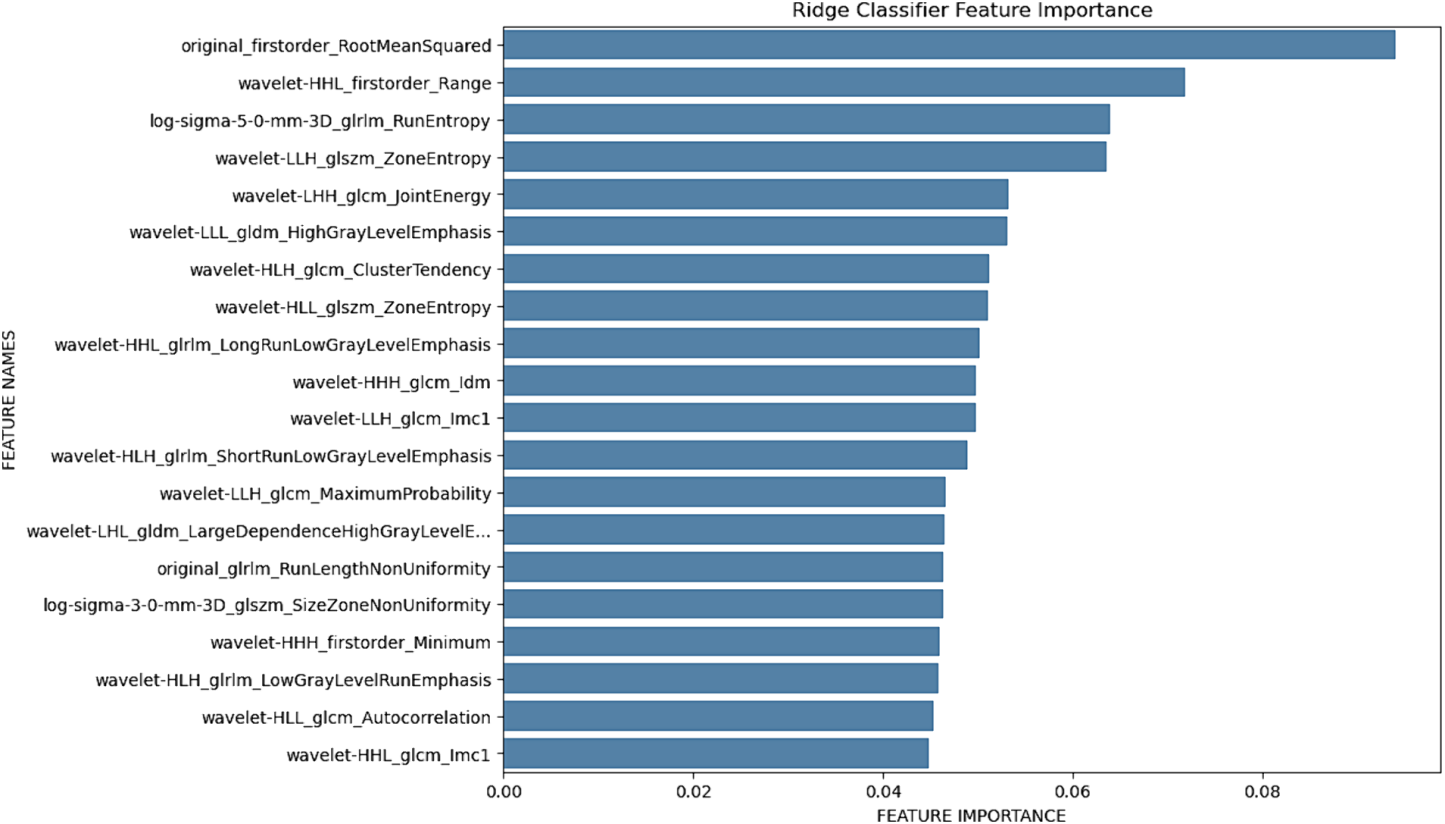
Histogram of important radiomics features for differentiation between phantom type calculated using Ridge regression.

Figure 3 shows the top 20 important radiomics features, listing wavelet- original_firstorder_RootMeanSquared as the most important one regarding the differentiation of plum and mandarin.

### CT scanner effectiveness

**Figure 4:**
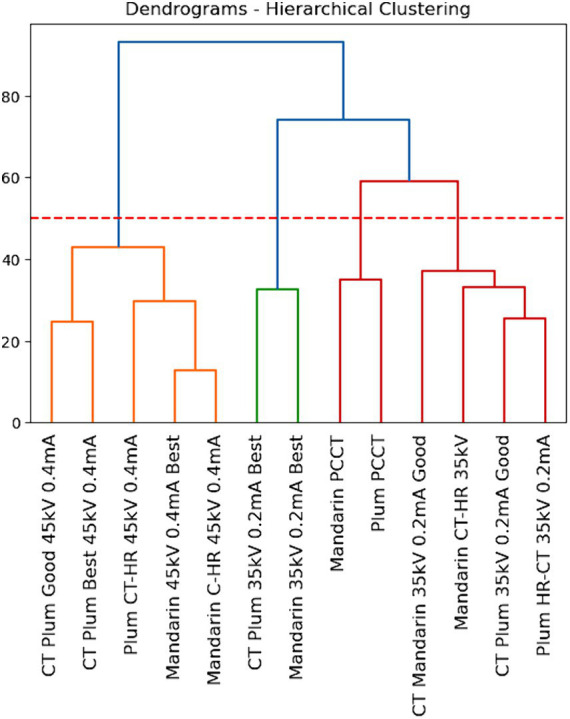
Dendrogram of dissimilarities between each cluster. The red line indicates the threshold of the dissimilarity to achieve the best number of clusters.

Simultaneously, the original data underwent agglomerative clustering, and a dendrogram plot was generated (Figure 4). The choice of a cutoff at 55 resulted in the formation of 4 distinct clusters. This clustering analysis revealed inherent patterns within the dataset, providing a comprehensive understanding of the underlying structure and relationships among data points. Figure 4 shows the dendrogram of the agglomerative clustering where the algorithm was able to cluster the data of PCCT scanner into one group, standard scanner with 45 keV into one group and 35 keV into another group. This clearly shows the difference in the quality of radiomics features extracted from these scanners.

The integration of both classification and clustering approaches enhances the interpretability of the results. The clusters identified through agglomerative clustering align with certain characteristics, potentially uncovering latent subgroups within the dataset indicating the type of scanner used. This multi-faceted analysis contributes to a more nuanced interpretation of the dataset, offering both classification accuracy metrics and clustering insights for a comprehensive understanding of the data’s complexity.

## Discussion

The presented study compared phantom scans (plums and mandarins) obtained from an experimental small animal PET/CT scanner and a Photon Counting CT scanner. The analysis involved varying scan settings to achieve different quality levels, and the resulting radiomics features were assessed using a Ridge Classifier. Additionally, agglomerative clustering was employed to explore inherent patterns within the dataset.

The results indicate that the experimental animal PET/CT scanner, particularly in “Best” and “High Resolution” modes, exhibited exceptional textural resolution. Taking into account the much faster scanning time and the significantly better dosing efficiency, excellent image resolution was also achieved with the scans on the Photon Counting CT. The Ridge Classifier, despite achieving a high training accuracy, faced challenges in generalization. The integration of classification and clustering approaches revealed distinct clusters corresponding to different CT scanners, highlighting the variability in radiomics features among these modalities.

Scale-independent and scale-dependent methods serve crucial roles in oncological imaging research and clinical practice, each offering distinct advantages and disadvantages that shape their impact on the broader landscape of this field. Scale-independent methods, exemplified by radiomics, excel in enhancing the transferability of research findings across different imaging modalities. By extracting quantitative features from medical images that are agnostic to the specific imaging device used, these methods facilitate the integration of data from diverse sources, promoting consistency and comparability in data interpretation. Moreover, scale-independent approaches expedite the translation of research insights into clinical practice by providing standardized frameworks for data analysis. However, they may overlook modality-specific nuances, potentially compromising the accuracy and reliability of clinical assessments. In contrast, scale-dependent methods are tailored to specific imaging modalities, optimizing performance within modality-specific contexts. They capitalize on the unique characteristics of individual imaging devices, maximizing sensitivity to scanner-specific features that may hold diagnostic or prognostic significance. Yet, their reliance on specific modalities limits their transferability across different types of imaging devices and makes them vulnerable to variability introduced by differences in imaging protocols and hardware configurations (24, 25).

In the broader context of radiomics, our findings underscore the potential of radiomics as a scale-independent translation tool between experimental and clinical CT scanners. The ability to differentiate scanners based on radiomics features aligns with previous studies emphasizing the modality-specific characteristics captured by radiomics (26). The challenges in generalization observed in our Ridge Classifier echo concerns raised in literature regarding the robustness of radiomic features across diverse datasets. This is a well-known problem, which is one of the main reasons why radiomics analyses have not yet found widespread use in clinical routine. In a previous study by Hertel et al., the test–retest stability of radiomics features was already examined in a phantom study using scans of the PCCT – the concordance correlation coefficient (CCC) of 0.9 demonstrated here indicates excellent test–retest stability (18).

There are several studies in the literature that have addressed the problem of the variability of radiomics parameters (27–29). Mackin et al. investigated the significance of inter-scanner variability of CT image-based radiomics studies (27). The features of tumors from NSCLC patients were compared with special phantoms consisting of different materials. In some cases, a large variability of the extracted parameters was found in the phantoms (depending on the respective materials), comparable to the variability that exists between the individual tumors of the different parameters.

Soliman et al. developed a model for the harmonization of radiomics parameters obtained from scans performed on different CT scanners (28). This allowed a reduction of scanner-associated variability of the data while preserving the cancer-specific functional dependence of the extracted features. Campello et al. investigated the radiomics variability of cardiovascular MRI datasets in a multi-centre study using a feature-based normalization technique (29). Feature distributions were initially compared across centers to derive a distribution similarity index. Two classification tasks were then conducted, showing that normalization of the radiomics parameters can effectively reduce variability with only a slight degradation in classification performance. Aside, piecewise linear histogram matching normalization produced features with improved generalization ability for classification.

However, an investigation of radiomics variability between the novel photon counting CT technique and experimental animal PET/CT scanners, which can achieve excellent spatial resolution with high radiation dose, has not yet been performed. This study is highly relevant with regard to clinical aspects and routine examinations of patients due to the translational aspects of animal models.

Despite the insightful findings, this study has certain limitations. The use of phantom scans may not fully replicate the complexity of *in vivo* conditions, and the generalization challenges encountered by the Ridge Classifier highlight the need for robust model training on diverse datasets. Additionally, the study focused on a specific set of radiomics features, and future work should explore a broader spectrum of features to enhance the robustness of the findings. In addition, the data set used was borderline small in terms of sufficient training of the classifier used. Furthermore, the radiomics stability is an issue because of the high multicollinearity between the corresponding features extracted (30). A linear model would easily run into the problem where the coefficients of the model become less significant and the model poorly performs with the test data. Reducing the dimensions by eliminating less important features could be one way to deal with the problem. LASSO exactly does this by shrinking the near zero coefficients to zero. However, LASSO does not consider features that are weakly correlated (31). Ridge regression on the other hand addresses this problem by not forcing the near zero coefficients to zero but penalizes the loss function by adding quadratic terms of coefficient values. This greatly reduces the impact of multicollinearity and thus stabilizes the coefficients (32).

The observed differences in radiomics features among scanners have important clinical implications. The identification of latent subgroups within the dataset based on scanner type suggests that these features could potentially influence diagnostic accuracy and treatment planning. Understanding the modality-specific characteristics revealed by radiomics may guide clinicians in selecting the most appropriate imaging modality for specific clinical scenarios, thereby improving patient outcomes. The results of examinations on animals using specialized scanners can be better understood and the information obtained can be translated more concretely to the diagnosis and treatment of humans.

In summary, our study contributes to the understanding of radiomics as a valuable tool for bridging the gap between experimental and clinical CT imaging. Our study shows that radiomics can be used as a statistical and thus scale-independent tool to enable a sufficient translation between animal CT scanners and human scanners (in this case the novel PCCT). Future directions should involve expanding the dataset to enhance model generalization, exploring additional radiomics features, and validating findings in clinical cohorts. A notable consideration in radiomics research is the high correlation and potential confounding effects among many radiomic features, as highlighted by Traverso et al. (33). They demonstrated that first-order features, such as entropy, tend to exhibit higher reproducibility compared to shape and texture metrics, which often show significant variability. This underscores the importance of carefully selecting and validating radiomic features to ensure robust and generalizable results. Our study, while focusing on a specific subset of features, acknowledges these findings and suggests that future work should not only expand the spectrum of investigated features but also incorporate rigorous methods to assess and mitigate feature redundancy and confounding effects, enhancing the reliability of radiomic analyses across different imaging modalities. As radiomics continues to evolve, its role as a scale-independent translation tool holds promise for improving the integration of experimental imaging technologies into clinical practice, ultimately advancing personalized medicine.

## Data availability statement

The raw data supporting the conclusions of this article will be made available by the authors, without undue reservation.

## Author contributions

AV: Conceptualization, Data curation, Formal analysis, Investigation, Methodology, Software, Supervision, Visualization, Writing – original draft, Writing – review & editing. CM: Writing – review & editing. FH: Writing – review & editing. FT: Writing – review & editing. HR: Data curation, Writing – review & editing. FP: Writing – review & editing. CW: Writing – review & editing. BW: Writing – review & editing. SS: Conceptualization, Supervision, Writing – review & editing. MF: Conceptualization, Data curation, Formal analysis, Investigation, Methodology, Supervision, Validation, Visualization, Writing – original draft, Writing – review & editing. AH: Conceptualization, Data curation, Formal analysis, Investigation, Methodology, Supervision, Validation, Visualization, Writing – original draft, Writing – review & editing.
